# The 5′ and 3′ Untranslated Regions of the Flaviviral Genome

**DOI:** 10.3390/v9060137

**Published:** 2017-06-06

**Authors:** Wy Ching Ng, Ruben Soto-Acosta, Shelton S. Bradrick, Mariano A. Garcia-Blanco, Eng Eong Ooi

**Affiliations:** 1 Programme in Emerging Infectious Diseases, Duke-National University of Singapore Medical School, Singapore 169857, Singapore; wyching.ng@duke-nus.edu.sg (W.C.N.); maragarc@utmb.edu (M.A.G.-B.); 2 Department of Biochemistry, University of Texas Medical Branch, Galveston, TX 77555, USA; rusotoac@utmb.edu (R.S.-A.); ssbradri@utmb.edu (S.S.B.); 3 Department of Microbiology and Immunology, Yong Loo Lin School of Medicine, National University of Singapore, Singapore 119077, Singapore; 4 Saw Swee Hock School of Public Health, National University of Singapore, Singapore 119077, Singapore; 5 Infectious Diseases Interdisciplinary Research Group, Singapore MIT Alliance in Research and Technology, Singapore 138602, Singapore

**Keywords:** Flavivirus, 3’untranslated region (3’UTR), subgenomic flaviviral RNA (sfRNA), viral replication

## Abstract

Flaviviruses are enveloped arthropod-borne viruses with a single-stranded, positive-sense RNA genome that can cause serious illness in humans and animals. The 11 kb 5′ capped RNA genome consists of a single open reading frame (ORF), and is flanked by 5′ and 3′ untranslated regions (UTR). The ORF is a polyprotein that is processed into three structural and seven non-structural proteins. The UTRs have been shown to be important for viral replication and immune modulation. Both of these regions consist of elements that are essential for genome cyclization, resulting in initiation of RNA synthesis. Genome mutation studies have been employed to investigate each component of the essential elements to show the necessity of each component and its role in viral RNA replication and growth. Furthermore, the highly structured 3′UTR is responsible for the generation of subgenomic flavivirus RNA (sfRNA) that helps the virus evade host immune response, thereby affecting viral pathogenesis. In addition, changes within the 3′UTR have been shown to affect transmissibility between vector and host, which can influence the development of vaccines.

## 1. General Features of Flaviviruses

Flaviviruses are arthropod-borne viruses that have been categorised into four groups: tick-borne flaviviruses (TBFV), mosquito-borne flaviviruses (MBFV), no-known vector flaviviruses (NKFV), and non-classified flaviviruses (NCFV). A more divergent genus known as insect-specific flaviviruses (ISFV) has also been recently identified [1,2]. TBFV and MBFV have been extensively studied, and as the names suggest, are transmitted through the bite of a tick or a mosquito, respectively, some of which cause serious illness in humans and animals. Some examples of viruses in these groups include tick-borne encephalitis virus (TBEV), dengue virus (DENV), West Nile virus (WNV), Japanese encephalitis virus (JEV), yellow fever virus (YFV), and Zika virus (ZIKV). 

Flaviviruses are enveloped viruses with a single stranded, positive sense RNA genome that is approximately 11 kb in size. Flaviviruses are taken up via receptor-mediated endocytosis by recognition of the envelope protein. Alternatively, flaviviruses such as DENV can opsonize with non- or sub-neutralizing levels of antibody, allowing entry into cells such as monocytes, macrophages, and dendritic cells via Fc gamma receptor-mediated endocytosis [3]. After virus internalization, the endocytic vesicle undergoes acidification, where fusion between the viral envelope and endosomal membrane occurs for the release of viral RNA into the cytoplasm to initiate viral replication. The single-stranded, positive-sense RNA genome serves as a messenger RNA, which is translated to generate viral proteins [4].

Flaviviral RNAs contain a type I cap at the 5′ terminus, but lack a polyA tail at the 3′ terminus. The RNA genome consists of a single open reading frame (ORF) that is flanked by 5′ and 3′ untranslated regions (UTRs) [5]. The ORF encodes a polyprotein which is cleaved by viral and host proteases, resulting in three structural proteins: capsid (C), pre-membrane/membrane (prM/M), and envelope (E), and seven non-structural (NS) proteins: NS1, NS2A, NS2B, NS3, NS4A, NS4B, and NS5. These proteins are cleaved by viral and host proteases during viral replication. Among the NS proteins, NS3 and NS5 are well characterized enzymatic proteins and are known to be major components of the replicative machinery [6]. The UTRs have been shown to be important for viral replication and immune modulation, and will be the focus of this review [7,8].

## 2. Flavivirus Untranslated Regions

**Figure 1:**
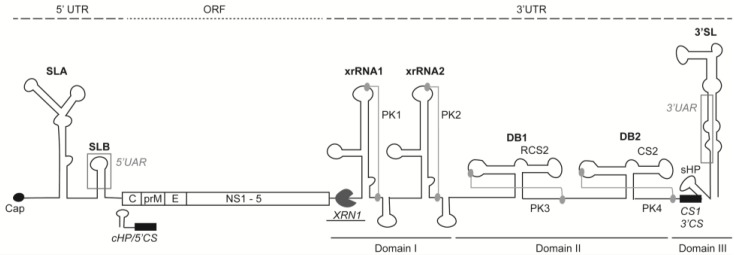
Schematic representation of dengue virus (DENV) genome. Location of the viral 5′ and 3′ untranslated region (UTR) and the open reading frame (ORF) indicating the structural (C-prM-E) and non-structural proteins (NS1-NS2A-NS2B-NS3-NS4A-NS4B-NS5). RNA structural elements located in the 5′ end includes the exoribonuclease XRN1 resistant RNA (xrRNA), stem loop B (SLB), 5′UAR (upstream AUG region), capsid-coding region hairpin element (cHP) and 5′CS (conserved sequence). The 3′UTR are defined into three domains: domain I (variable region) consists of xrRNA1 and xrRNA2, strengthened by pseudoknots (PKs) 1 and 2. Domain II incorporates dumbbell structures DB1 and DB2, which contain repeated conserved sequence 2 (RCS2) and CS2 sequences together with PK3 and PK4. Domain III is the most conserved region with short hairpin structure (sHP), CS1, 3′UAR, and 3′CS, which makes up the 3′SL (stem loop). XRN1 stalls at the first stem loop, as indicated. In the case of DENV4, where xrRNA1 is missing, XRN1 stalls at xrRNA2. C: capsid; E: envelope; prM: pre-membrane.

The flavivirus genome is flanked by 5′ and 3′UTRs. The non-coding 5′UTR of flaviviruses spans around 100 nucleotides in length, whereas the 3′UTR ranges from 400 to 700 nucleotides in length, depending on the virus species. The interaction between 5′ and 3′UTRs are critical for viral RNA replication. These interactions are necessary for the recruitment and correct positioning of the NS5 RNA polymerase for initiation of minus strand RNA synthesis [9,10,11,12]. Many studies have identified RNA elements within the UTRs that are essential for flavivirus translation, replication, and pathogenesis in mammalian and mosquito cells [10,13,14,15,16]. These include the 5′ stem loops A and B (5′SLA and 5′SLB, respectively), 5′ and 3′ upstream AUG region (UAR), 3′ cyclization sequence, 3′ short hairpin structure (sHP), the highly-conserved 3′SL and the 5′ cyclization sequence, and the capsid-coding region hairpin element (cHP) that lies within the ORF (Figure 1) [10,12,17,18,19]. Both the 3′SL and the 5′SLA are crucial, as removal of either structure completely abolished virus production [20,21].

The cHP element that lies within the coding region is conserved among flaviviruses, and has been shown to aid in start codon recognition and viral replication. Mutations to this element abrogated WNV and DENV replication, as cHP may function to recruit or stabilize initiation factors [19]. It was shown that basepairing that restored stem formation in mutant cHP elements had no effect on viral replication, leading to the conclusion that cHP function is sequence-independent but structure-dependent [22]. On the other hand, the 3′ sHP is responsible for mediating a balance between linear and circular forms of the genome [23]. Furthermore, the 3′ sHP has been shown to be important for viral replication, as mutations in the sHP have been shown to be lethal for DENV in mosquito cells [24].

Genome cyclization is necessary for RNA synthesis, and it requires several complementary regions between the 5′ and 3′UTR to form a panhandle—namely, the 5′–3′ cyclization sequence and 5′–3′ UAR sequences [25,26]. The 5′ cyclization sequence is located within the ORF, while the 3′ cyclization sequence is located immediately upstream of the 3′SL. Both the 5′ and 3′ cyclization sequence are made up of eight nucleotides, and the degree of base pairing has been shown to affect the rate of replication [25,27,28]. Although originally found to be eight nucleotides in length, it was later shown to be 10, 11, and 18 nucleotides (encompassing the eight core nucleotide) for DENV, WNV, and YFV [9,29]. Mutation of the 5′ and 3′ cyclization motifs showed that complementarity rather than exact sequence is more important for panhandle formation leading to viral RNA replication [25]. In addition, there is a flavivirus-conserved penta-nucleotide 5′-CACAG-3′ sequence on the top loop of 3′SL that is also crucial for RNA replication. A study with the Kunjin virus replicon system showed that the first and last nucleotide of the five nucleotide sequence is essential for Kunjin virus replication [30]. This was also observed with YFV and WNV, although point mutations to 2nd, 3rd, and 4th bases can have varying effects on viral replication [31,32,33].

The 5′UAR is located within SLB, and 3′UAR overlaps the sHP at the bottom of the 3′SL (Figure 1). These complementary sequences were shown to be essential for successful DENV and WNV replication [34,35]. In addition to these two complementary sequences, a recent study showed that another pair of complementary segments—termed downstream of AUG region (DAR)—are also important [36]. The 3′DAR sequence is located within the sHP region of the 3′SL. Both sHP integrity and 5′–3′ DAR complementarity were found to be essential for WNV RNA replication [37]. While the cyclization sequence is highly conserved among flaviviruses, the 5′DAR sequence is only conserved among all four DENV serotypes, but not with other flaviviruses such as WNV, Kunjin virus, and JEV [36].

## 3. 5′ Untranslated Region

The 5′UTR is short, with around 100 nucleotides upstream of the capsid gene. This region contains a m^7^GpppAmpN1 cap structure and two conserved stem-loop regions—SLA and SLB (Figure 1) [12,38,39]. The cap structure in flavivirus genomes is thought to be important for cap-dependent translation and protection from cellular 5′–3′ exonucleases [40,41]. However, DENV is also able to translate by a poorly understood cap-independent mechanism when cap-dependent translation is inhibited [42]. The SLA and SLB are approximately 70 and 30 nucleotides in length, respectively. They are separated by a poly(U) sequence to allow for proper functioning of SLA and SLB that is required for RNA synthesis and does not have a role in genome cyclization [12]. SLA is the promoter for RNA synthesis, which activates NS5 polymerase to initiate RNA synthesis at the 3′ end of the circular genome [11,18]. SLB sits closest to the ORF start codon, and contains the indispensable 5′UAR sequence needed for long range RNA–RNA interaction for genome replication [29]. Furthermore, a highly-conserved RNA duplex region termed 5′-UAR-flanking stem (UFS) has been identified and was shown to interact with NS5 for the initiation of RNA synthesis: mutations in the UFS abrogated flavivirus replication [43]. 

Although the 5′UTR sequences are variable among flaviviruses, the RNA structure is mostly conserved, with some variation between strains of MBFV and TBEV [44]. Nonetheless, for both groups of viruses, this region is critical for genome cyclization, viral RNA synthesis, and translation [45,46]. Due to the critical function of the 5′UTR, studies have examined the sequence conservation within each of the four DENV serotypes. In DENV3, it was observed that although the 3′UTR showed some degree of variation, the 5′UTR was highly conserved [47]. This observation was also seen with four clinical isolates of DENV2 from patients in Thailand, showing that the 5′UTR was identical to the much older New Guinea C strain [48]. Furthermore, only one nucleotide change was observed in the 5′UTR of DENV2 16,681 and its attenuated derivative, PDK53. However, this C57U nucleotide change, together with single nucleotide changes in the NS1 and NS3, appear to be functionally important in conferring the attenuated phenotype: PDK53 produces small plaque size, reduced replication in C6/36 mosquito cells, and decreased neurovirulence in mice [49,50]. The C57U mutation in the 5′UTR was later shown to be reversible after repeated passages of PDK53 in Vero cells, suggesting the significance of 5′UTR sequence conservation for viral fitness [51]. 

## 4. 3′ Untranslated Region

The flaviviral 3′UTR ranges in length from ~400 to 600 bases, and is highly structured with regions conserved between flaviviruses. It is made up of stem-loop (SL) and two dumbbell (DB) structures and within these structures lie conserved sequences (CS) and repeated conserved sequences (RCS) [27,52]. The length of 3′UTR varies depending on the species of virus, but it is mainly divided into three domains: domain 1 immediately downstream of the stop codon consists of a variable nucleotide sequence between arthropod-borne flaviviruses containing 2 SL structures, which have been referred to in different terms (Table 1); domain 2 is moderately conserved, with two DB structures (DB1 and DB2) incorporating RCS2 and CS2; and domain 3 is highly conserved across flavivirus groups and it contains the CS1, small hairpin (sHP), and a large terminal 3′SL structure (Figure 1). The 3′SL is structurally conserved among flaviviruses, and is functionally important. It interacts with host and viral proteins to modulate viral RNA synthesis and translation [53,54,55,56,57,58]. In between major structures in the 3′UTR, there are spacer sequences which may promote proper folding and prevent interference between structural domains. The upstream RNA structures are important for the maintenance of conformational structure necessary for optimal viral replication [59].

The length of the domain 1 variable region (VR) varies between arthropod-borne flaviviruses, and the functional reason for this variation is currently unknown. Repeat sequences are found in the VR, and spontaneous deletions have been observed during serial passaging. In fact, deletion in the VR of the TBEV genome increased the virulence of this virus in mice [60,61]. In addition, Alvarez et al. observed that the VR in DENV2 altered viral growth in mammalian but not mosquito cells [10]. More recently, using evolutionary conservation analysis, the sequence and structure of SL structures in the variable region that are resistant to host exoribonuclease (XRN1) activity—or xrRNA1 and xrRNA2—were shown to be highly conserved during replication in mammalian but not mosquito cells, suggesting varying functions of such structures in different hosts [62]. 

The SL and DB structures of 3′UTR are further stabilized by pseudoknots (PK) [55,63,64]. The top loop of both DB (5′ GCUGU 3′) pairs with a complementary motif within the A-rich sequence between the structures to form two sequential PKs, and these PKs have been shown to be important for viral RNA synthesis [65]. The DB structures also play a role in translation, as deletion of both DB structures reduced translation by 60% [66]. Although DBs are involved in translation, they play a more significant role in RNA replication and the formation of the sfRNA (see below) [66,67].

Direct repeats of CS/RCS (which are found on the DBs) are 20–45 nucleotides in length that are known to be mostly conserved in flaviviruses [27]. CS1 and CS2 are found in DENV, JEV, and YFV, whereas RCS2 is found only in DENV and JEV. The NKVF group also shares the CS2 and RCS2 domains [68]. CS3 and RCS3 are only found in JEV subgroups, while three tandem repeats of YF-R1, YF-R2, and YF-R3 are found in YFV subgroups [69]. These conserved sequences could have evolved as regions that interact with cellular proteins to maximize replication for increased transmission in different hosts [70]. Although it was thought that the sequence in CS/RCS must be essential due to its duplication, multiple groups have shown that such repeat sequences are redundant, as removal of one CS/RCS did not majorly alter virus fitness [71,72,73,74]. However, this observed redundancy may be a laboratory phenomenon that may not accurately represent evolution in nature. Sequence alignment have shown that flavivirus 3′UTR evolved by duplications of an ancient RNA motif that is related to a repeat sequence in TBFV [63,75]; duplicated direct repeats could hasten the assembly of replication complex, since host and viral proteins responsible for RNA synthesis exist as dimers. This suggestion is further strengthen by the fact that YFV strains in West Africa have tandem repeats while those in Central/East Africa or South America do not, possibly due to the need for triple DRs in a specific reservoir host in West Africa [70]. Furthermore, the 3′SL of WNV have been shown to bind host protein T-cell intracellular antigen-1 (TIA-1) and TIA-1-related protein TIAR; knockdown of TIA-1/TIAR negatively affected viral replication [76]. Additionally, mutations in the region where WNV 3′SL binds TIA proteins were known to affect RNA synthesis [77]. TIA-1/TIAR host proteins also mediate the translation of DENV and TBEV RNA [77,78]. Binding of these host proteins to flaviviruses’ 3′UTR could thus either help stabilize the replication complex or help in the polymerase recognition or recruitment.

Over the years, multiple studies have attempted to identify and determine the region of 3′UTR that is critical for viral replication. Viruses made via reverse genetic techniques that delete distinct but limited regions of the 3′UTR have provided us with information regarding the necessity of the 3′UTR nucleotide sequence and RNA structures for virus replication [71,79,80]. Replicon systems have also been used to show that certain mutations or deletion of the 3′UTR results in reduced replication rate [72,81]. Furthermore, the luciferase replicon system was used to show that low-passage Nicaraguan DENV isolates were less able to translate viral proteins, possibly due to differences in the 3′UTR nucleotide sequence compared to its precursor Thai 16681 wild-type strain [82]. This is in line with a study done by Proutski et al., where a genetic algorithm was used to simulate the folding of a modified sequence of DENV4 3′UTR to determine the effects of structural rearrangements [59]. The authors observed that the length of sequence deletion is inversely correlated with infectivity, whereby longer deletions caused less reduction in infectivity, suggesting that the structure rather than sequence that is important for the 3′UTR function. A recent study showed an association between specific SLs and viral fitness, suggesting strong evolutionary pressure to maintain those RNA structures [62].

## 5. Generation of sfRNA

In addition to the critical role 3′UTR plays on RNA synthesis, the RNA structures have also been shown to be important for stalling the host XRN1 exoribonuclease to form sfRNA. Subgenomic flavivirus RNA was first characterized in Murray Valley encephalitis viral infections, followed by JEV and WNV; it was later substantiated in YFV, DENV, and more recently, Zika [83,84,85,86,87]. sfRNA is approximately 0.5 kb in size, and is thus far shown to be produced by incomplete degradation of RNA by the host 5′–3′ exoribonuclease XRN1 activity. XRN1 is responsible for mRNA degradation in actively dividing cells, and digests uncapped monophosphorylated mRNA in a 5′ to 3′ direction [88]. sfRNA is produced due to the stalling of XRN1 by xrRNA1/xrRNA2 in the 3′UTR. Recent structural models of xrRNA derived from X-ray diffraction data revealed the folding of these RNA structures that can explain XRN1 stalling [86,89]. A recent study showed that different species of sfRNA are produced depending on the host: the majority of sfRNA found in human cells is produced by exoribonuclease stalling at xrRNA1, whereas stalling in mosquito appears to be predominantly in the DB structures [90]. 

The folding of xrRNA1 is critical for XRN1 resistance, and the PK that stabilizes the helical arrangement further prevents XRN1 from unwinding the RNA [91]. Mutations or disruption of PK1 and PK2 in WNV were shown to disrupt the generation of sfRNA, producing smaller species of sfRNA which in turn attenuated the virus [67,84]. This was also observed in Zika, where disruption of PK severely impaired XRN1 activity [87]. The redundancy of both xrRNA1 and xrRNA2 for sfRNA production was suggested to ensure sufficient production; notably, xrRNA1 is absent in DENV4. Alternatively, xrRNA1 and xrRNA2 may play different roles under different intracellular conditions [24]. 

sfRNA has multiple roles, including effects on cytopathology in cell culture and viral pathogenesis in mice, suggesting its role in disease outcome [84,92]. sfRNA was shown to modulate the immune response by affecting RNA interference (RNAi) mechanisms, mRNA turnover pathways, and type-I interferon response [92,93,94,95,96]. In insects, the highly abundant sfRNA has been postulated to act as decoy molecules for RNAi mediators Dicer and Ago2 to prevent it from cleaving dsRNA molecules [92]. In mammalian cells, it is known that sfRNA acts as an antagonist of both interferon and retinoic acid-inducible gene-I (RIG-I)-dependent innate immune response [81,94]. sfRNA can also affect antiviral responses by serving as binding site for cellular proteins such as G3BP1, G3BP2, and CAPRIN that are needed for translation of interferon-induced mRNAs [97]. 

Studies on mosquitoes during Kunjin virus infection of *Culex quinquefasciatus* mosquitoes showed that sfRNA mildly suppressed the RNAi response, resulting in decreased virus transmission without compromising mosquito fitness [98]. The authors suggested that sfRNA causes a reduced small interfering RNA (siRNA)-mediated response, as it interferes with the loading of siRNA onto the RNA induced silencing complex (RISC) [98]. Furthermore, a recent publication using sfRNA-deficient WNV infection of mosquitoes showed that the generation of sfRNA is crucial for infection and transmission, because sfRNA helped WNV bypass the midgut barrier to enable high viral titres in the saliva [99]. These are all important observations, as arboviruses must replicate to a sufficient titer in the saliva without affecting mosquito fitness for onward transmission to mammalian or avian hosts. Although shutting down the RNAi pathway is important to bypass the midgut barrier, excessive inhibition of RNAi during DENV2 infection was shown to be lethal for mosquitoes [100,101,102]. Therefore, a balance must reached for vector survival and efficient replication of flaviviruses for onward transmission. 

A recent study by Chapman et al. compared xrRNA1 and xrRNA2 for XRN1 stalling activity for sfRNA production [86]. Kieft et al. then proposed that when DENV cycles through mosquitoes and humans, the 3′UTR mutates to regulate the amount of sfRNA produced—less sfRNA is produced in mosquitoes, but upon interaction with humans, the 3′UTR mutates to produce more sfRNA [89]. This observation was also shown by Villordo et al., whereby DENV variants that favour RNA and PK structures that interfere with XRN1 activity were selected in mammalian cells [62]. 

## 6. Vaccine Implications

The antibody-dependent enhancement (ADE) hypothesis which has been a widely used explanation for the clinical association between secondary DENV infection and increased risk of severe dengue, was recently demonstrated clinically in a trial [103]. ADE occurs when non- or sub-neutralizing levels of antibodies opsonize DENV to augment the infection of Fc-receptor-expressing cells to increase viral and antigenic burden for deleterious pro-inflammatory responses that exacerbate pathology. Therefore, the development of a live-attenuated DENV vaccine must induce strong protective immunity against all four serotypes, which has proven to be challenging. Many advances have been tried over the years, including alterations in the 3′UTR. 

This was first demonstrated when a deletion of 30 nucleotides (Δ30) within domain II in the 3′UTR of DENV4 was found to attenuate infection in rhesus monkey [71]. A study by Durbin et al. further proved that DENV4Δ30 mutant was attenuated in humans, as it was well tolerated in healthy volunteers who received this candidate vaccine [104]. The same mutation was later introduced into the same region of DENV1 [105] and DENV2 [106], where both were shown to be attenuated in rhesus monkeys, with DENV2Δ30 being the less attenuated strain. With DENV3, Δ30 deletion did not confer any sort of attenuation in severe combined immune deficiency (SCID) mice transplanted with human hepatoma cells (SCID-HuH7), monkeys, or mosquitoes, unlike the other serotypes [107]. Therefore, to derive an attenuated DENV3 vaccine candidate, the authors generated a chimeric virus with the DEN4Δ30 genomic backbone spliced with DENV3 prM/E genes (rDEN3/4Δ30). This chimeric virus was shown to be both attenuated and immunogenic in rhesus monkeys, although the phenotype was attributed to chimerisation rather than deletion within the 3′UTR [107]. Moreover, viruses with the Δ30 mutation were unable to disseminate from the midgut to the salivary glands of mosquito, which is a desirable trait for a vaccine candidate [108]. Although initial results for DEN3Δ30 was discouraging, further studies with additional mutation located 55 nucleotides upstream of the original Δ30 sequence (rDEN3Δ30/31) showed attenuation in SCID-HuH7-mice and rhesus monkeys [109]. A further study used these viruses to formulate a tetravalent vaccine that produced seroconversion to all four DENV serotypes [110]: A human DENV challenge study showed that immunization with TV003 vaccine (which is composed of rDEN1Δ30, rDEN2/4Δ30, rDEN3Δ30/31, and rDEN4Δ30) was able to prevent disease development [111]. More recently, a 10-nucleotide deletion of the 3′UTR of ZIKV resulted in a strain of virus that showed potentially useful properties of attenuation [112].

The use of chimeric viruses has been useful in generating DENV vaccines. Genomes of vaccine strains PDK53 or YF17D have served as backbones to deliver the prM/E genes of DENV1, DENV2, DENV3, and DENV4. Curiously, a tetravalent dengue vaccine (TDV) that uses PDK53 genome as backbone and chimeric yellow fever/dengue vaccine (CYD-TDV) that uses a YF17D backbone produced highest level of protection against DENV2 and DENV4, respectively. It is tempting to speculate that an interplay between the prM/E and 3′UTR region could be an important consideration for optimized vaccine candidates [113,114]. Interplay between prM/E and 3′UTR was observed when chimeric DENV2 viruses were made with substitution of the E, 5′UTR, and/or 3′UTR between South-East Asian and American genotype: a more pronounced reduction in viral replication was observed when replacement of the 5′UTR or 3′UTR was accompanied by mutation in the E gene [80]. Furthermore, a chimeric virus that combines the structural genes of TBEV with DEN4Δ30 was more attenuated when mutations were also introduced in both the TBEV E and DENV4 NS5 genes compared to just changing the 3′UTR alone [115]. Therefore, it could be useful to examine if there are specific interactions between the 3′UTR and the structural genes or proteins.

## 7. Conclusions

The untranslated regions of flaviviruses serve important functions for virus replication and survival. These highly structured regions play an important role in forming the pan-handle structure for genome circularisation required for RNA synthesis and viral translation. The 5′UTR is highly conserved among flaviviruses, pointing to the importance of this region for virus replication. In addition to mediating viral replication, the 3′UTR also assists the virus in evading host innate immune responses, hence contributing to infection outcome in the vector and/or non-vector hosts. Furthermore, the variable region in the 3′UTR allows for changes to occur that enable virus adaptation as it switches between vector and non-vector hosts. Detailed functional understanding of the flaviviral UTRs could provide further insights into viral evolution, fitness, and vaccine design.

## Figures and Tables

**Table 1:** Nomenclature used to describe 3′ untranslated regions (UTR) stem loop structure of dengue virus (DENV).

Downstream of Stop Codon:				
1st SL	2nd SL	1st DB	2nd DB	Reference
SL II	SL IV			[62]
DEN-SLI	DEN-SLII			[90]
xrRNA1	xrRNA2	xrRNA3	xrRNA4	[84,92]

DB: Dumbell structures; SL: stem loop; DEN: dengue; xrRNA: XRN1 Resistant RNA
